# Caspase-1 Activation via Rho GTPases: A Common Theme in Mucosal Infections?

**DOI:** 10.1371/journal.ppat.1000795

**Published:** 2010-02-26

**Authors:** Andreas J. Müller, Claudia Hoffmann, Wolf-Dietrich Hardt

**Affiliations:** Institute of Microbiology, D-BIOL, ETH Zürich, Zürich, Switzerland; The Fox Chase Cancer Center, United States of America

## Caspase-1 Activation: More Common among Enteropathogens than We Thought?

Caspase-1 is an important converging point for danger signals initiating inflammation and defense. Recent work suggests that RhoGTPase activation and/or cytoskeletal disturbance may represent a novel pathway eliciting caspase-1 responses that are subverted by several enteropathogenic bacteria. The enteropathogen *Salmonella* Typhimurium employs the type III effector protein SopE, an activator (guanine nucleotide exchange factor [GEF]) for RhoGTPases, to elicit caspase-1 maturation and release of the pro-inflammatory cytokine IL-1β to trigger gut inflammation in vivo. Recently, a whole new family of pathogen-encoded RhoGTPase GEFs has been discovered, including Map, IpgB1, and IpgB2 from enteropathogenic *Escherichia coli* and *Shigella* spp.. We will discuss the evidence suggesting that these virulence factors may also activate caspase-1 signaling.

## Caspase-1 Integrates Multiple Danger Signals

**Figure 1:**
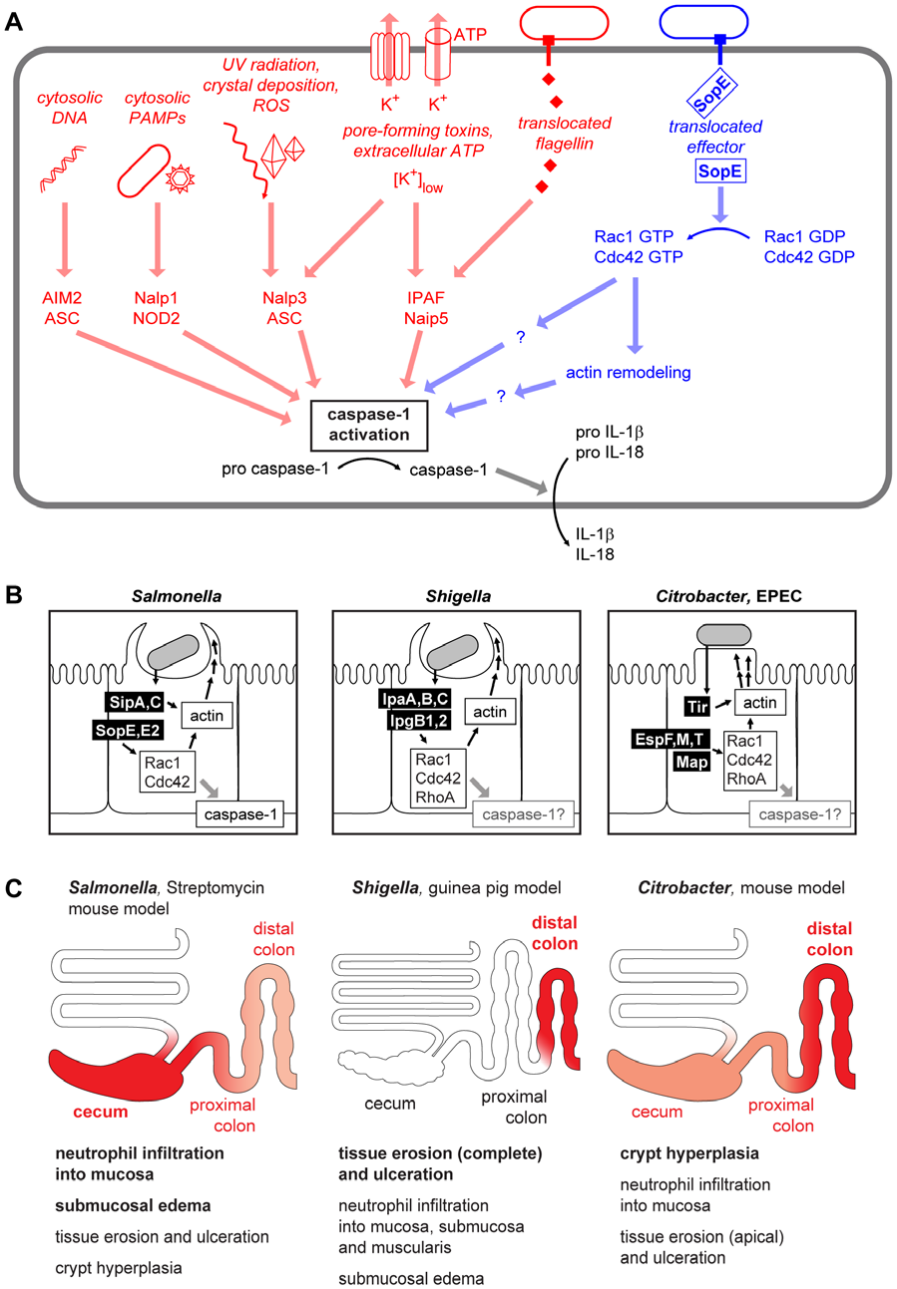
Danger-associated signaling via the inflammasome. (A) Cytosolic microbe-associated molecular patterns and danger signals are detected and integrated by an array of inflammasome components converging at the activation of caspase-1 [5], [38]–[40]. In addition to the already known stimuli for inflammasome activation (left, red), a pathway involving *S.* Typhimurium SopE and Rho GTPases (right, blue) has been described recently [8]. (B) Comparison of the virulence mechanisms of *Salmonella* spp., *Shigella* spp., and *Citrobacter*/EPEC. The effector proteins injected can act both directly on the actin polymerization as well as via the activation of Rho GTPases [41]–[43]. (C) Comparison of pathologies in different animal models of *Salmonella*, *Shigella*, and *Citrobacter*/EPEC infection. The region of the strongest pathology and the most important pathological changes in each model is indicated in red. Representative images for the macroscopic and histological changes in the respective animal models can be found in references [44]–[48].

Mucosal surfaces are constantly exposed to a large number of commensal, non-pathogenic microorganisms towards which the gut immune system remains non-responsive [1]. In the case of an acute infection, this homeostasis is overruled and an inflammatory response is initiated. Detection of pathogen-derived molecules, bacterial growth, or other “danger signals” indicating infection or trauma occurs via “pattern recognition receptors” and other detectors on the surface and in the cytosol of mucosal cells and most other cells of the body [2],[3]. This detection process induces the production of pro-inflammatory cytokines, which initiate the innate immune defense. Caspase-1 represents an important converging point for processing danger signals: physical stress, pore-forming toxins, extracellular ATP, and the presence of conserved bacterial products (e.g., flagellin or peptidoglycan) in the cytosol are detected via danger-sensing molecules and adaptors [4]. These bind to caspase-1 and lead to the formation of multiprotein complexes called inflammasomes [5]. Thereby, caspase-1 is activated and catalyzes the maturation and release of pro-inflammatory cytokines like interleukin (IL)-1β and IL-18 (Figure 1A). Thus, caspase-1 activation is a central regulator of the innate immune defense. Recent work has indicated that the activation of RhoGTPases, in particular Rac1 and possibly Cdc42, might represent a novel type of signaling input that can activate caspase-1 signaling and which may represent a point of attack for bacterial virulence factors (Figure 1A, right, [6]–[8]).

## SopE, a Potent Activator of Host Cellular RhoGTPases

SopE from the enteropathogenic bacterium *Salmonella enterica* subspecies I serovar Typhimurium (*S*. Typhimurium) is a well-known functional mimic of mammalian GEFs [9]. SopE is delivered into host cells via the bacterial type III secretion system-1 (TTSS-1), binds to host cellular RhoGTPases, including Rac1 and Cdc42, and activates them by catalyzing rapid G-nucleotide exchange [9],[10].

The activity of cellular RhoGTPases depends on guanine nucleotide binding and is regulated by various cellular proteins. Rho-specific cellular GEFs stimulate the nucleotide exchange on RhoGTPases. Upon release of GDP and subsequent binding of GTP, the RhoGTPase switches to its active conformation and is now able to signal to downstream effectors [11]. By mimicking the function of endogenous GEFs, SopE disturbs the RhoGTPase activation cycle and initiates signaling downstream of Rac1 and Cdc42, respectively [9].

**Figure 2:**
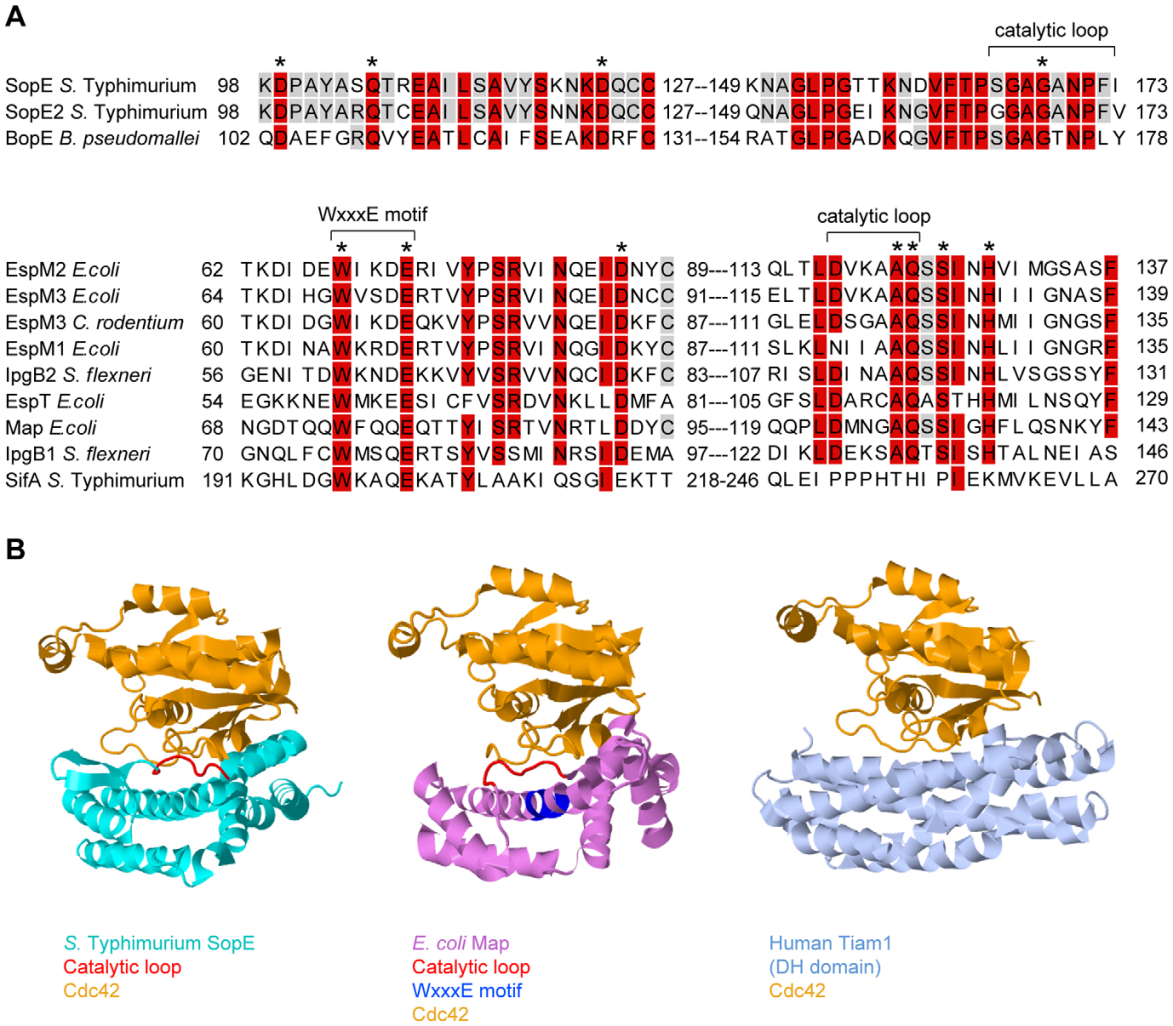
Sequence and structural comparison of GEFs from different origins. (A) Sequence alignment of SopE-type (upper alignment) and WxxxE-type (lower alignment) bacterial effector proteins with postulated Rho GTPase activity; >80% identity is shaded in red, >50% in grey. The residues shown to be important for catalytic GEF activity of SopE [13] or Map [19] are marked with asterisks. WxxxE motif and catalytic loops highlighted in (B) are marked with brackets. Note that SifA has a completely different amino acid sequence in its catalytic loop compared to the other proteins in the WxxxE family [19]. (B) Crystal structures of GEFs from different organisms in complex with Cdc42 (orange). Left panel: SopE (cyan) from *S.* Typhimurium [12]; the catalytic loop is highlighted in red. Middle panel: Map (pink) from enteropathogenic *E. coli* [19]; the WxxxE motif is highlighted in blue and the catalytic loop in red. Right panel: Human Tiam1 (pale blue); only the Rho GTPase binding DH domain is shown [14]. Structures were created using Jmol (http://www.jmol.org/).

SopE is the prototype of a family of bacterial RhoGTPase GEFs that also includes SopE2 and BopE from *Salmonella* and *Burkholderia* spp. (Table 1, Figure 2A). In spite of the functional similarity, SopE-like GEFs have an entirely different three-dimensional structure than the Dbl-like mammalian GEFs for RhoGTPases (Figure 2B, [12]). SopE is composed of two three-helix bundles and a small GA-rich loop in the catalytic center. Nonetheless, several amino acid contacts with the RhoGTPase and the basic catalytic mechanism are shared with Dbl-like mammalian GEFs [12],[13]. Moreover, both GEF families bind to the same surface of the RhoGTPase, i.e., the “switch 1” and “switch 2” regions that facilitate G-nucleotide binding [12],[14]. Thus, SopE-like GEFs of pathogenic bacteria are “functional mimics” of host cellular Dbl-like GEFs.

**Table 1:** Guanine Nucleotide Exchange Factors Involved in Pathogenesis and/or Caspase-1 Activation.

Effector	Pathogen/Organism	Host Model System (for In Vivo Phenotype)	Activated Target GTPase^a^	Caspase-1 Activation Shown	Pathology	Virulence Defect of GEF Mutant		Reference
						In Vitro	In Vivo	
***SopE-type bacterial GEFs***								
BopE	*Burkholderia pseudomallei*	Murine systemic infection	Cdc42, Rac1		Abscess formation (melioidosis)^b^	+	+	[49]–[51]
SopE	*Salmonella enterica*	Murine enterocolitis, bovine ileal loops	Rac1, Cdc42	+	Gut inflammation	+	+	[8],[9],[15],[17],[31],[52]
SopE2	*Salmonella enterica*	Murine enterocolitis, bovine ileal loops	Cdc42 (Rac1)	+^c^	Gut inflammation	+	+	[8], [17], [52]–[54]
***WxxxE-type bacterial GEFs***								
EspM1^d^	Enteropathogenic *Escherichia coli*		Unknown		Attaching/effacing lesions	+		[55]
EspM2/3^d^	*Citrobacter rodentium,* enteropathogenic *Escherichia coli*		RhoA		Attaching/effacing lesions	+		[55]
EspT^d^	Enteropathogenic *Escherichia coli*		Rac1, Cdc42		Attaching/effacing lesions	+		[56]
IpgB1	*Shigella flexneri*	Murine lung infection	Rac1 (Cdc42)		Bacterial dysentery	+	+^e^	[19],[21],[57]
IpgB2	*Shigella flexneri*	Murine lung infection	RhoA (Rac1, Cdc42)		Bacterial dysentery	+	+^e^	[19],[20],[57]
Map^f^	*Citrobacter rodentium*, enteropathogenic *Escherichia coli*	Murine enterocolitis	Cdc42		Attaching/effacing lesions	+	+	[19],[20],[58],[59]
SifA^d^	*Salmonella enterica*		RhoA		Gut inflammation	+		[60]
***Dbl-type eukaryotic GEFs***								
Tiam1	Human		Rac1 (Cdc42)	+^c^				[8],[61]

a For some effector proteins, the Rho GTPase specificity has not been analyzed in sufficient detail; therefore, specificity for additional Rho GTPases not listed is likely for some of the GEFs.

b *B. pseudomallei* causes a wide variety of disease symptoms among different hosts, including pneumonia, gut inflammation, and diarrhea [62].

c Upon transfection.

d Sequence homology data and GEF activity not directly shown.

e IpgB1 and IpgB2 were shown to cooperatively contribute to the in vivo phenotype.

f *C. rodentium* and enteropathogenic *E. coli* share highly homologous forms of Map [63]. The in vivo phenotype was shown with *C. rodentium* Map, whereas GEF activity was shown experimentally with *E. coli* Map.

## Caspase-1 Activation by SopE

SopE is one of the key TTSS-1 effectors driving host cell invasion and the induction of mucosal inflammation by *S*. Typhimurium [9], [15]–[17]. SopE was shown to induce a number of host cell responses, including the activation of JNK and PAK signaling and prominent actin cytoskeleton rearrangements [9],[18]. Therefore, it has been assumed that SopE- and RhoGTPase-mediated tissue invasion and/or JNK or PAK activation was sufficient to explain how SopE contributes to gut inflammation. However, recent work has identified a different biological mechanism: SopE-mediated RhoGTPase activation was found to drive not only tissue invasion, but also caspase-1 activation and the release of IL-1β (Figure 1A, right [8]). Caspase-1 was also activated in cells over-expressing constitutively active Rac1, constitutively active Cdc42, or an active mutant of the Dbl-like Rac1-GEF Tiam1 [6],[8]. Moreover, knockout mice lacking caspase-1/IL-1/IL-18 responses still allowed SopE-mediated tissue invasion, but failed to mount mucosal inflammation. In bone marrow chimaeric mice, caspase-1 was required primarily within stromal cells of the gut mucosa, most likely enterocytes [8]. Together, these data indicated that GEF-induced RhoGTPase activation in the mucosal epithelium can elicit caspase-1/IL-1β/IL-18-dependent gut inflammation.

## A New Family of Bacterial GEFs

Recently, a second family of bacterial type III effector proteins, the WxxxE family, was found to possess GEF activity for host cellular RhoGTPases [19]. The WxxxE family includes Map from enteropathogenic *E. coli* (EPEC), IpgB1 and IpgB2 from *Shigella*, and SifA and SifB from *Salmonella* spp.. These proteins harbor a unique flexible loop in the catalytic site and an invariant WxxxE motif (Figure 2A). Although phylogenetically unrelated to SopE-like GEFs, the WxxxE family GEFs display a protein fold that is highly similar to that of SopE/E2 and employ the same catalytic mechanism (Figure 2B). Several WxxxE family members, e.g., Map, IpgB1, and IpgB2, have been shown to induce RhoGTPase activity [19]. Interestingly, they differ in their substrate specificities (Table 1, [19],[20]). For example, in vivo, IpgB1 activates Rac1 and Cdc42 [21], whereas IpgB2 preferentially activates RhoA, but also Cdc42 and Rac1 in vitro [19]. This observation suggests that, depending on the preferred target, at least a subset of WxxxE GEFs might serve similar functions as SopE in pro-inflammatory signaling.

## Caspase-1: An Achilles' Heel Subverted by Mucosal Pathogens?

The vast majority of SopE or WxxxE family type III effector proteins have been detected in enteropathogenic bacteria (Table 1). In the case of SopE, GEF-induced RhoGTPase activation was shown to elicit caspase-1 responses and the release of caspase-1-dependent cytokines, i.e., IL-1β and IL-18, thus leading to gut inflammation [8]. Based on these data, we hypothesize that the WxxxE family members found in enteropathogenic *E. coli* and *Shigella* spp. may have the same biological function, i.e., activating caspase-1 in the host's intestine. Among these effector proteins, GEFs with specificity for Rac1 and Cdc42 are especially good candidates to activate caspase-1, since activated Rac1 (and to a lesser extent Cdc42) has been shown to activate caspase-1 [6]. Thus, GEF-mediated caspase-1 activation might be a common strategy of enteropathogenic bacteria for enhancing mucosal inflammation (Figure 1B). This is of special significance in light of earlier work on *Salmonella* and *Citrobacter* infection models showing that enteropathogenic bacteria can benefit from eliciting gut inflammation: although some aspects of the pathology differ between the model systems (e.g., proximal/distal location of the most severe lesions in the large intestine Figure 1C), the host's inflammatory response is thought to suppress growth of the competing commensal microflora in either case [22]–[24]. Therefore, it is tempting to speculate that virulence factors with Rac1- and/or Cdc42-specific GEF activity might allow enteropathogens to subvert Rho GTPase-mediated caspase-1 activation in order to gain an edge against the commensal microflora.

## Analysis of Caspase-1 Activation by Bacterial Effector Proteins—A Tricky Task

In principle, knockout mice deficient in caspase-1, IL-18, or IL-1 signaling allow the analysis of caspase-1 activation by candidate virulence factors. However, this analysis is tricky for two different reasons.

First, caspase-1 activation and IL-1/IL-18 signaling represent an important arm of the innate immune defense (Figure 1A) which helps to clear pathogens and limits further pathogen-inflicted damage [25]–[29]. Therefore, caspase-1, IL-1, or IL-18 deficiency tends to increase pathogen loads in infected tissues, can trigger additional (caspase-1/IL-1/IL-18 independent) pro-inflammatory pathways and amplify disease symptoms. This phenotype has been observed in *Shigella* and *Citrobacter* infection experiments and may have masked WxxxE protein-induced effects [25],[30].

Second, there is appreciable functional overlap between the *Shigella*, *Salmonella*, and EPEC virulence factors inducing mucosal inflammation. Not all of them require caspase-1 [8],[31]. Therefore, the contribution of caspase-1-dependent virulence factors may be “masked” in wild-type infections, as was shown to be the case in *Salmonella* infections [8],[28]. The investigation of virulence factors activating caspase-1 thus requires knowledge about functionally overlapping pro-inflammatory mechanisms. These would have to be disrupted in order to unequivocally identify (or refute) a caspase-1-mediated virulence mechanism.

Thus, in the case of pathogens employing SopE or WxxxE family effector proteins, caspase-1, IL-1, or IL-18 deficiency should have two opposing effects: reducing the responsiveness towards the caspase-1-activating virulence factor versus increasing pathogen loads (and possibly damage) in the host's tissue. Therefore, the experiments need to be designed carefully in order to discern these opposing effects. For example, SopE-mediated caspase-1 activation was clearly observed in *S.* Typhimurium strains lacking other virulence factors, but it was masked in the context of the wild-type pathogen [8],[28]. A similar balance between caspase-1-mediated innate defense and pathogen-induced pathology may have prohibited unequivocal detection of WxxxE protein-mediated caspase-1 activation in the cases of *Shigella* spp., *Citrobacter* spp., or enteropathogenic *E. coli* [30]. Pathogen mutants stripped of all but the caspase-1-dependent virulence factor protein, effector protein expression in a *Salmonella* mutant stripped of all relevant effector proteins, or tissue culture transfection experiments might help to circumvent this technical problem.

## The RhoGTPase–Caspase-1 Connection: What's in Between?

In HEK293 cells, Rac1 and Cdc42 activation is sufficient for activating caspase-1 [6],[8]. However, it has remained unclear whether a specific downstream effector protein of Rac1 and/or Cdc42 or disturbance of the actin cytoskeleton inflicted by the activated RhoGTPases transmits the danger signal towards caspase-1. The Rac1 effector kinase LIMK was suggested to be involved in signaling to caspase-1 [6], and direct interaction of caspase-1 with and phosphorylation by PAK-1 was shown [32]. Other reports support a role for actin and actin-binding proteins. The gelsolin family protein flightless-I binds and inhibits caspase-1, thereby possibly linking caspase-1 activity to the actin cytoskeleton [33]. In macrophages, inflammasome components might be sequestered by perinuclear F-actin structures that form upon certain inhibitory stimuli [34]. The molecular links between Rac1/Cdc42 and inflammasome activation remain to be elucidated.

## An Evolutionary Role for Linking Actin Polymerization to Caspase-1 Activation

The detection of microbe-associated molecular patterns alone is not sufficient to induce an inflammatory response in the gut mucosa. Additional signals characteristic of pathogen growth within a tissue or other trauma are thought to be required before the innate immune system responds. A prominent example of such a two-layered control mechanism is the pro-inflammatory cytokine IL-1β. Pro-IL-1β expression is induced upon TLR4 stimulation by bacterial lipopolysaccharide (LPS). However, the pro-IL-1β has to be cleaved and activated by caspase-1 before it is secreted [35]. Caspase-1 in turn is activated in response to numerous danger signals [36]. Manipulation or disruption of the cell cytoskeleton has been proposed recently as a “pattern of pathogenicity” signal that feeds into caspase-1 activation and pro-IL-1β processing [37]. Based on this, it is tempting to speculate that Rac1/Cdc42 activation or actin disturbance itself might represent a pathogen-associated danger signal sensed by the innate immune system. If this held true, the SopE and WxxxE families of type III effector proteins might subvert a pathogen-sensing mechanism for eliciting mucosal inflammation. With knockout mice deficient in caspase-1 and several inflammasome components and a growing number of useful infection models at hand, investigating the involvement of WxxxE GEFs in caspase-1 activation will be an interesting task for future research.

## Accession Numbers

The UniProt (http://www.uniprot.org/uniprot/) accession numbers for the protein sequences used for alignment in Figure 2A are O52623 (*S. enterica* sv. Typhimurium SopE), Q7CQD4 (*S. enterica* sv. Typhimurium SopE2), Q63K41 (*B. pseudomallei* BopE), C6URF9 (*E. coli* EspM2), B5YYM1 (*E. coli* EspM3), B1GVN9 (*C. rodentium* EspM3), Q8VQ34 (*E. coli* EspM1), Q9AJW7 (*S. flexneri* IpgB2), B9WN88 (*E. coli* EspT), Q7DB76 (*E. coli* Map), Q6XVY7 (*S. flexneri* IpgB1), and Q56061 (*S. enterica* sv. Typhimurium SifA).

The PDB (http://www.rcsb.org/pdb/) accession codes for the structures shown in Figure 2B are 1gzs for SopE [12], 3cgc for Map [19], and 1foe for Tiam1 [14].
